# Successful pilot application of multi-attribute utility analysis concepts in evaluating academic-clinical partnerships in the United States: a case report

**DOI:** 10.3352/jeehp.2022.19.18

**Published:** 2022-08-19

**Authors:** Sara Elizabeth North, Amanda Nicole Sharp

**Affiliations:** Division of Physical Therapy, Medical School, University of Minnesota, Minneapolis, MN, USA; Hallym University, Korea

**Keywords:** Feasibility studies, Health occupations, Partnership, Evaluation, Physical therapy, Case report

## Abstract

Strong partnerships between academic health professions programs and clinical practice settings, termed academic-clinical partnerships, are essential in providing quality clinical training experiences. However, the literature does not operationalize a model by which an academic program may identify priority attributes and evaluate its partnerships. This study aimed to develop a values-based academic-clinical partnership evaluation approach, rooted in methodologies from the field of evaluation and implemented in the context of an academic Doctor of Physical Therapy clinical education program. The authors developed a semi-quantitative evaluation approach incorporating concepts from multi-attribute utility analysis (MAUA) that enabled consistent, values-based partnership evaluation. Data-informed actions led to improved overall partnership effectiveness. Pilot outcomes support the feasibility and desirability of moving toward MAUA as a potential methodological framework. Further research may lead to the development of a standardized process for any academic health profession program to perform a values-based evaluation of their academic-clinical partnerships to guide decision-making.

## Introduction

### Background

Strong partnerships between academic health professions programs and clinical healthcare practice settings, termed academic-clinical partnerships, are essential in providing quality full-time clinical training experiences [1,2]. Unfortunately, challenges in contemporary clinical education are increasingly identified by scholars and experts in the field. For example, the clinical education model in Doctor of Physical Therapy (DPT) education was deemed by a 2017 national task force as “unsustainable, suboptimal, and not designed to produce practitioners required by the health care system of the future” [3]. Key challenges consistently reported for 50 years include unwarranted variability in clinical practice sites, inconsistent clinical education quality, burdensome student performance evaluations, economic restrictions, and diminishing capacity to accommodate full-time learners [3-5].

Decreased availability of student placement slots may require academic programs to increase their volume of clinical partners. A growing quantity of clinical partners reduces the ability of an academic program to maintain, enhance, and evaluate quality partnerships. Interactions between partners are often suboptimal, triggered by issues needing resolution rather than a drive for mutual engagement [6,7]. To address this issue, it is recommended that educational researchers explore partnership frameworks designed to promote effective communication and evaluation practices [1,3,5].

What is unclear, however, are the precise conditions and factors that promote strong academic-clinical partnerships. Such constructs have not been operationalized to date and there is no model by which an individual program may assess its priorities. The concept of partnerships remains undefined as well, referencing both legal affiliation agreements and operational relationships guiding placement processes. The inherent variability in academic program missions, resources, operations, student clinical education needs, and academic faculty skills and experiences suggests that partnership priorities are likely not the same for every program.

### Objectives

This case report aims to present one academic DPT clinical education program’s pilot implementation of a values-based academic-clinical partnership evaluation approach, rooted in methodologies from the field of evaluation.

### Ethics statement

Study subjects were not human but programs; therefore, neither approval by the institutional review board nor obtainment of the informed consent is necessary.

## Case presentation

To establish a baseline, the authors as clinical education faculty members in the DPT program at the University of Minnesota began with an informal assessment of their existing partnerships. A subjective ‘grade’ of A, B, C, or D was assigned to each partner based on interactions with that partner from 2015 to 2020 (Supplement 1). Partners earning a grade of A, or ‘excellent’, were perceived as consistent in meeting program needs, reliable, and provided high quality experiences. Partners earning a grade of B, or ‘good’, were perceived as somewhat consistent, somewhat reliable, and provided moderate quality experiences. Partners earning a grade of C, or ‘poor’, were perceived as inconsistent, often unreliable, and provided fair to poor quality experiences. Partners earning a grade of D, or ‘unacceptable’, demonstrated minimal to no placement history and had poor reliability and/or placement quality. This served as a brainstorming exercise in generating an initial conceptualization of partner characteristics perceived as contributing to effective partnerships in practice.

The authors then sought a way to quantitatively evaluate partnership factors to determine the extent to which existing clinical partners supported the program’s priority values. These values were aligned with the academic program’s mission, vision, and operational resources. Because health professions literature did not offer a systematic process to perform this evaluation, the team explored methodological processes in the field of evaluation. The faculty identified an evidence-based decision-making framework with potential called multi-attribute utility analysis (MAUA) [8,9] (Supplement 2). This framework facilitates comparative analysis of multiple alternatives with unique complex attributes. In the absence of a single measure of effectiveness, the methodological process may be used to identify and quantify the decision maker’s preferences and values through the execution of five prescribed procedural steps. Priority attributes can then be ranked to simplify the process and reduce inconsistencies in decision-making. While successful application of MAUA as an objective evaluation process has been demonstrated in a variety of fields, including public health, law, and private-public city project partnerships, utility analysis as a measure of effectiveness has been minimally used by educational researchers to date [8].

As a pilot study, the academic clinical education faculty opted to incorporate evaluation principles associated with MAUA to assess the feasibility and desirability of conducting a full MAUA for partnership prioritization in a future study. None of these steps had been implemented in partnership literature to date.

First, the authors considered partnership literature, program faculty perspectives, institutional context, and professional experience to generate their priority partnership list. Ten attributes were selected as follows: setting (e.g., inpatient placements), level variety (early versus advanced learners), frequency (over a year), location (e.g., rural sites), state (in- versus out-state), relationship with clinical education team (e.g., loyalty, strong communication), relationship with program/university (e.g., alumni, lab assistants), relationship with students (e.g., high site ratings), administrative burden (on program), and student burden (additional costs, time). Each attribute was operationalized as noted with parenthetical examples.

Next, the direct method was used as described in MAUA to assign importance weights to each attribute by allocating a total of 20 points among the 10 prioritized attributes based on their perceived relative importance [8]. An iterative process ensued as allocations were refined through criterion clarification and weighted balance adjustments in initial partner evaluation attempts.

Third, the additive approach was used to calculate the total utility, or effectiveness, of each partnership by summing the points for all attributes. The final point system indicated 20 as the highest partnership effectiveness score and 0 as the lowest. An institution-specific final rubric was created, termed the Clinical Partner Prioritization Rubric (CPPR) (Supplement 3).

**Fig. 1.**
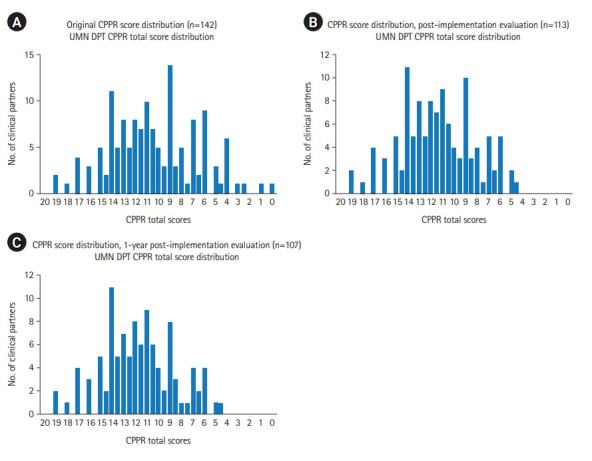
Clinical Partner Prioritization Rubric (CPPR) total score distributions at original (A), post-implementation (B), and 1-year post-implementation (C) time points. UMN DPT, University of Minnesota Doctor of Physical Therapy.

**Fig. 2.**
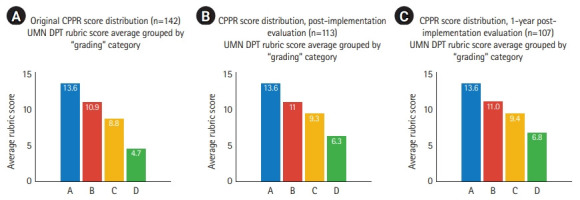
Clinical Partner Prioritization Rubrice (CPPR) score averages grouped by “grading” category at original (A), post-implementation (B), and 1-year post-implementation (C) time points. UMN DPT, University of Minnesota Doctor of Physical Therapy.

An initial CPPR score was determined for 142 distinct clinical partners (Fig. 1A), with a mean score of 10.38/20. The clinical education faculty compared partners’ initial CPPR scores with original subjective “grades” to informally assess CPPR construct validity (Fig. 2A). A linear relationship demonstrated that mean CPPR scores were higher for partners with positive subjective grades (A: mean=13.6; B: mean=10.9) and lower for partners with poorer subjective grades (C: mean=8.8; D: mean=4.7).

The clinical education team used CPPR data to guide initial actions. The team reviewed partners with the lowest CPPR scores and determined whether attribute utilities were changeable or unchangeable. For those with multiple unchangeable circumstances (e.g., site location), affiliation agreements were terminated, and the sites were removed from the partner list. For those with changeable circumstances (e.g., frequency of placement offers for first rotation learners), meetings were scheduled to discuss partnership growth opportunities. The team also reviewed partners with the highest CPPR scores and developed strategies to strengthen collaborative placement planning and clinical faculty training.

**Fig. 3.**
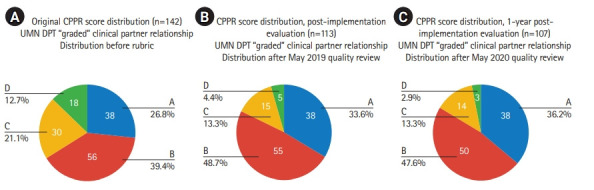
Proportion of “graded” clinical partner relationships at original (A), post-implementation (B), and 1-year post-implementation (C) time points. CPPR, Clinical Partner Prioritization Rubric; UMN DPT, University of Minnesota Doctor of Physical Therapy.

These actions taken following the first CPPR analysis resulted in a revised CPPR mean score of 11.46/20 and a reduction in total partners evaluated from 142 to 113 (Fig. 1B). Again, this volume represents distinct partners identified for CPPR evaluation purposes, with some partnerships incorporating multiple facility locations. Aggregate subjective grade data also improved (Fig. 2B), with the proportional volume of partners graded as C or D decreasing from 33.8% of partners to 17.7% (Fig. 3A, B).

The CPPR evaluation was completed again one year later. Actions following this CPPR analysis resulted in an aggregate CPPR mean score of 11.66/20 and a reduction in total partners evaluation from 113 to 107 (Fig. 1C). In the first year of CPPR implementation, the number of affiliated clinical partner relationships scoring less than 6/20 on the CPPR reduced from 9.86% (14/142) to 1.9% (2/107), which served as the self-selected benchmark for partnership stratification. The proportion of partners graded as C or D decreased from 17.7% to 16.2% (Figs. 2C, 3C).

Raw response data are available from Dataset 1.

## Discussion

Pilot outcomes support the feasibility and desirability of moving toward MAUA as a potential methodological framework for evaluating academic-clinical partner relationships. The semi-quantitative evaluation approach developed in this pilot incorporated evaluation concepts rooted in MAUA methodology, successfully enabling academic clinical education faculty to create and consistently apply a values-based prioritization process specific to the institutional context.

Over the course of one year, data-informed actions resulted in the elimination of lower-scoring academic-clinical partnerships and increased relationship-building with highly aligned clinical partners, leading to improvement in overall partnership effectiveness. Continued research may lead to the development of a standardized process by which any academic health profession program could perform a values-based evaluation of their academic-clinical partnerships to guide decision-making in partner relations. Further research is needed to assess the utility of MAUA for academic-clinical partnership evaluation over time and across institutional contexts. Questions remain regarding comparisons of priority partnership attributes across academic programs and professions, optimal frequency of partner evaluation, cutoff scores to objectively stratify levels of partnership effectiveness, and potential for a variant of this process for clinical partners to evaluate their priority values in academic-clinical partnerships.

Findings in this case study begin to address national clinical education challenges cited in the literature. Further exploration of strategies and structures supporting strong academic-clinical partnerships is needed to advance knowledge regarding the conditions that promote strong clinical training of health professionals.

## Footnotes

**Data availability**
Data files are available from Harvard Dataverse: https://doi.org/10.7910/DVN/S3BNNZ. Dataset 1. Raw data of participants’ response to the rubric, Clinical Partner Prioritization Rubric.

## Supplementary materials

Data files are available from Harvard Dataverse: https://doi.org/10.7910/DVN/S3BNNZ.

Supplement 1. Data for figures in excel format.

Supplement 2. Multi-attribute utility analysis (MAUA) steps.

Supplement 3. Clinical Partner Prioritization Rubric (CPPR).

Supplement 4. Audio recording of the abstract.
