# Supplementary material for: Successful pilot application of multi-attribute utility analysis concepts in evaluating academic-clinical partnerships in the United States: a case report
Source: J Educ Eval Health Prof. 2022 Aug 19;19:18. doi: 10.3352/jeehp.2022.19.18 (PMC9534602; doi:10.3352/jeehp.2022.19.18)
Supplement: Supplementary file 3 — Supplement 2. Multi-attribute utility analysis (MAUA) steps. [file jeehp-19-18-suppl2.docx]

**Supplement 2.** Multi-attribute utility analysis (MAUA) steps [8,9]

| The 5 steps of MAUA facilitate comparative analysis of multiple alternatives with unique complex attributes |
| --- |
| 1. Operationalize priority valued elements in decision making |
| 2. Determine single utility (value) of each attribute |
| 3. Assign importance weights to each attribute |
| 4. Calculate weighted utility of each attribute |
| 5. Additive approach to calculate total utility of an option |
