# Supplementary material for: Successful pilot application of multi-attribute utility analysis concepts in evaluating academic-clinical partnerships in the United States: a case report
Source: J Educ Eval Health Prof. 2022 Aug 19;19:18. doi: 10.3352/jeehp.2022.19.18 (PMC9534602; doi:10.3352/jeehp.2022.19.18)
Supplement: Supplementary file 4 — Supplement 3. Clinical Partner Prioritization Rubric (CPPR). [file jeehp-19-18-suppl3.pdf]

PHYSICAL THERAPY
UNIVERSITY OF MINNESOTA
Driven to Discover℠

Clinical Partner Prioritization Rubric

This tool is designed to support a culture of excellence in physical therapy clinical education by quantitatively identifying the degree to which clinical partner relationships serve the program's prioritized full-time clinical education needs.

1) Subjectively grade each clinical partner or site using an ABCD scale based on the perceived academic-clinical relationship over the past 5 years. Refer to grading guidelines on the next page.

2) Score each clinical partner or site using the rubric criteria and scoring guidelines. Award no points if guideline items do not apply. Refer to action response stratification on the next page.

	Partner #1	Partner #2	Partner #3
Grade (ABCD)			

Criteria	Possible Points	Scoring guidelines	Partner #1	Partner #2	Partner #3
1. Setting (25%)	5	5 - Inpatient<br>4 - Specialty (e.g. pediatric, pelvic health, lymphedema)<br>4 - Uniqueness (e.g. federal prison, professional athletics)<br>3 - Meets program requirement (e.g. OP neuro, TCU)			
2. Variety of Placement Levels (15%)	3	1 - CE I only<br>1.5 - CE I plus one other level<br>2 - CE I plus two other levels<br>1 - CE II, III, and IV, not including CE I<br>3 - All four CE levels			
3. Frequency (5%)	1	1 - Places 3+ students per year			
4. Location (10%)	2	2 - Rural sites * Choose only one<br>1 - Local metro sites			
5. State location (5%)	1	1 - Within the state			
6. Relationship with clinical education team (10%)	2	1 - Loyalty, reliability<br>1 - Strong SCCE communication<br>(-1-2) - Deduction: Poor SCCE comm. (difficult, untimely)<br>(-1-2) - Deduction: Repeated admin. burden on academic prog. (re-sending links, forgetting confirmed placements)			
7. Relationship with Program/University (10%)	2	1 - Alumni presence<br>1 - Consistent clinical employees with didactic connection (e.g. lab assistants, guest speakers)			
8. Relationship with students (10%)	2	2 - High student ratings on APTA PTSE<br>(-2) - Deduction: Low student ratings on APTA PTSE			
9. Clinical education team administrative burden (5%)	1	1 - Minimal admin time needed for contract management<br>(-1) - Deduction: Additional logistics/admin req. for placement, completed by academic program			
10. Student burden (5%)	1	1 - Site offers free housing<br>(-1) - Cost of additional requirements (e.g. out of state background check, lab tests)			
Total possible:	20	Total score:			

© 2019 Sara North PT, DPT, M.Ed. and Amanda LaLonde PT, DPT

PHYSICAL THERAPY
UNIVERSITY OF MINNESOTA
Driven to Discover℠

Clinical Partner Prioritization Rubric

Subjective partner relationship grading guidelines

Grade	Quality of partner relationship
A	Excellent- Consistently meets program needs, reliable, high quality experience
B	Good- Somewhat consistent, somewhat reliable, moderate quality experience
C	Poor- Inconsistent, often unreliable, fair to poor quality experience
D	Unacceptable- Minimal to no placement history, poor reliability and/or quality

Action Response Stratification

Score	Action response
16-20	Enhance and maintain relationship
11-15.5	Nurture relationship development
6-10.5	Reassess use of partner in the future
0-5.5	Discontinue partnership

© 2019 Sara North PT, DPT, M.Ed. and Amanda LaLonde PT, DPT
